# Mixture prioritization and testing: the importance of toxicokinetics

**DOI:** 10.1007/s00204-021-03026-y

**Published:** 2021-03-17

**Authors:** Albert Braeuning, Philip Marx-Stoelting

**Affiliations:** 1 Department of Food Safety, German Federal Institute for Risk Assessment, Max-Dohrn-Str. 8-10, 10589 Berlin, Germany; 2 Department of Pesticides Safety, German Federal Institute for Risk Assessment, Max-Dohrn-Str. 8-10, 10589 Berlin, Germany

The topic of mixture toxicity has gained increased awareness in the past few years. Toxicological risk assessment is still mainly based on the analysis of individual compounds, while the consideration of mixture effects is already stipulated in some cases, e.g. in the field of pesticides. Given the almost infinite number of chemicals and their combinations, there is a need to develop strategies for grouping compounds and for the prioritization of potentially critical mixtures, which might exert effects that deviate from the general assumption of dose addition.

This may be achieved by approaches, which classify chemicals according to their toxicological properties, as, for example, established for pesticides: here, the active compounds are grouped into so-called common assessment groups (CAGs) (EFSA 2008, 2009, 2013). Even though these CAGs consider different aspects of hazard and do thus also consider some aspects of toxicokinetics, they set a strong focus on toxicodynamics. Common target organs or mechanisms of action, as they e.g. form the basis of the CAGs, are frequently used as a basis for the selection of chemicals for mixture testing. One example is the EU-funded project EuroMix: within that project, methodologies and tools for mixture risk assessment have been developed anchored in the adverse outcome pathway (AOP) concept and mixture selection mainly based on the toxicodynamic profiles of the test chemicals (Alarcan et al. 2021; Beronius et al. 2020).

Martin et al. (2021) have recently published a systematic review article on experimental analysis of chemical mixtures. The authors conclude that the dose addition concept appears to be suitable for most mixtures, while nonetheless the potential of some classes of chemicals to produce non-additive, especially synergistic mixture effects, should not be disregarded. Interestingly, the authors show that many published synergistic effects relate to metabolic, i.e. toxicokinetic interference. Of note, this is in accordance with observations from the pharmaceutical field demonstrating that pharmacokinetic interference is a major cause of undesired drug-drug interactions (Martin et al. 2021).

In line with the notion that toxicokinetic differences appear to play a relevant role as the underlying cause of non-additive effects, a recent paper by Lasch et al. demonstrates that triglyceride accumulation in human liver cells caused by a triazole-class fungicide can be exacerbated by the presence of an additional, non-steatotic compound, and that this effect is related to an interference of the second compound with the cytochrome P450-mediated metabolism of the fungicide (Lasch et al. 2021). The findings presented in the paper highlight the importance of toxicokinetic interactions as a major cause of non-additive mixture effects. Moreover, the fact that the combination of a steatotic compound with a non-steatotic compound was able to amplify the observed cellular steatotic effect also highlights possible limitations of a mixture prioritization strategy solely relying on common toxicodynamic properties of the mixture constituents.

CAG- and AOP-based approaches are undoubtedly useful tools for grouping and mixture selection. Nonetheless, it appears that by focusing on toxicodynamic characteristics, the importance of toxicokinetic interferences as a mechanistic basis for non-additive mixture effects tends to be disregarded and underrated. Therefore, it appears timely that categorization of compounds, besides CAG grouping or similar strategies, should additionally be performed on a toxicokinetic basis, covering e.g. induction and inhibition of metabolic enzymes as well as interference related to effects at transport proteins. Therefore, it is proposed to develop a novel grouping approach based on common kinetic groups (CKG), to identify, select and prioritize compounds and their mixtures, which could interact at the toxicokinetic level leading to toxic effects beyond the assumption of additivity.
